# Evolution of proteins involved in the final steps of juvenile hormone synthesis

**DOI:** 10.1016/j.jinsphys.2023.104487

**Published:** 2023-03

**Authors:** Vlastimil Smykal, David Dolezel

**Affiliations:** a Biology Center of the Academy of Sciences of the Czech Republic, Institute of Entomology, Ceske Budejovice, Czech Republic; b Faculty of Science, University of South Bohemia, Ceske Budejovice, Czech Republic

**Keywords:** Juvenile hormone, Methyl transferase, Epoxidase, Evolution, Gene duplication, Alternative splicing

## Abstract

Juvenile hormone (JH), a sesquiterpenoid produced by the insect *corpus allatum* gland (CA), is a key regulator of insect metamorphosis, reproduction, caste differentiation, and polyphenism. The first part of JH biosynthesis occurs via the universal eukaryotic mevalonate pathway. The final steps involve epoxidation and methylation. However, the sequence of these steps might not be conserved among all insects and Crustacea. Therefore, we used available genomic and transcriptomic data and identified JH acid methyltransferase (JHAMT), analyzed their genomic duplications in selected model organisms, and reconstructed their phylogeny. We have further reconstructed phylogeny of FAMeT proteins and show that evolution of this protein group is more complicated than originally appreciated. The analysis delineates important milestones in the evolution of several JH biosynthetic enzymes in arthropods, reviews major literature data on the last steps of JH synthesis, and defines questions and some hypotheses worth pursuing experimentally.

## Highlights

• Final steps of juvenile hormone (JH) synthesis differ among insects.
• Evolution of the last enzymes in JH biosynthesis was reconstructed.
• JH methyltransferase (JHAMT) gene undergoes frequent duplications.
• JHAMT duplications are usually gene or genus-specific.

## Graphical abstract

**Figure:**
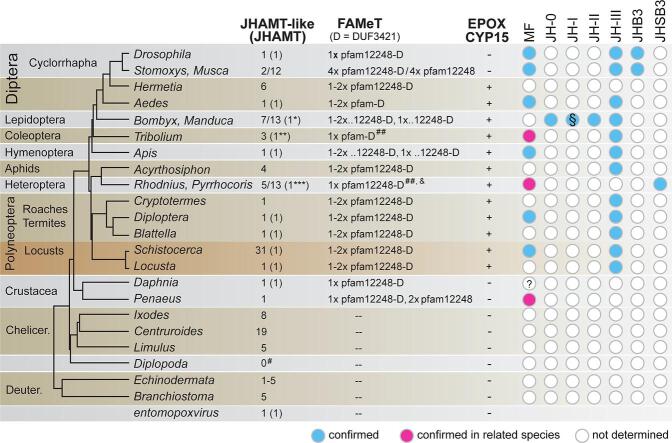

## 1 Introduction

One of the greatest teachers we met during our undergraduate years was Professor František Sehnal, who not only spent a fair amount of time explaining the treacherous aspects of developmental biology to us but also enjoyed introducing us to the basics of insect taxonomy and evolution. Given this tireless educational effort and his lifelong scientific interest in juvenile hormone (JH), we would like to dedicate this small study to his memory.

In Wigglesworth's classical studies, JH was shown to be a key hormone in preventing metamorphosis of the nymphal stages to adults (Wigglesworth, 1934, Wigglesworth, 1936, Wigglesworth, 1940). The role of JH was further expanded when JH was found to play a key role in reproduction, to be involved in caste differentiation of social insects such as termites, to contribute to polyphenism, and to influence behavior (Nijhout, 1994, Hartfelder and Emlen, 2012). Since JH was identified even in ametabolous insects, the role in reproduction is evolutionarily older than its role in metamorphosis (Sehnal et al., 1996). JH is synthesized in the *corpora allata* (CA), a neurohemal gland from where it is distributed to target tissues via the hemolymph (Wigglesworth, 1948, Tobe and Pratt, 1974). The reception of JH was deciphered relatively recently. The key protein of the JH receptor is Methoprene tolerant (MET), a member of the basic Helix-Loop-Helix Per-ARNT-Sim (bHLH-PAS) family of transcription factors (Ashok et al., 1998). Upon exposure to JH, MET forms complexes with another bHLH-PAS protein TAIMAN, and together they trigger reproduction in males and females (Smykal et al., 2014; Marchal et al., 2014; Urbanova et al., 2016, Holtof et al., 2021). MET is required for JH to prevent precocious metamorphosis, as shown for holometabolous species (Konopova and Jindra, 2007), and hemimetabolous insects (Konopova et al., 2011). The interaction of MET with another bHLH-PAS protein CYCLE has been identified biochemically in yeast assays (Shin et al., 2012). JH-dependent reprogramming of gut expression in the linden bug *Pyrrhocoris apterus* requires MET and two circadian clock proteins CYCLE and CLOCK, both of which belong to bHLH–PAS family (Bajgar et al., 2013).

**Fig. 1:**
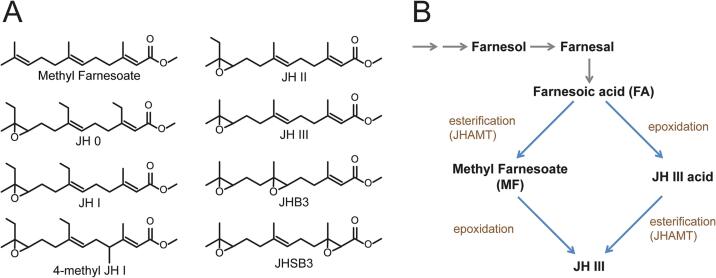
Juvenile hormones and the last steps of their biosynthesis. (A) Methyl Farnesoate (MF) and seven types of Juvenile hormone (JH) are detected in insects. (B) Last steps proposed for JH biosynthesis in insects (modified from Wen et al., 2015, Minakuchi et al., 2015). The mevalonate pathway leads to the synthesis of Farnesoic acid (FA) from which JH III is produced by two alternative pathways. In one scenario, FA is methylated by Juvenile Hormone Acid Methyl Transferase (JHAMT) leading to MF from which JH III is produced by epoxidation. In the alternative biosynthetic pathway, FA is first epoxidized to JH III acid which is then methylated by JHAMT. CYP15 is the known epoxidase involved in the above-mentioned epoxidation steps; however, in some species, additional epoxidases may be involved. Also, the synthesis of JH in true bugs (Heteroptera), which contain a second epoxide group, is not included in this scheme. For further details, see the text.

The chemical structure of the first JH types was identified in the 1960s using the silkworm *Hyalophora cecropia* (Röller et al., 1967, Meyer et al., 1968). Gradually, other JH types were discovered, so that today there are seven recognized types of naturally occurring JH (Fig. 1A). Chemically, all insect JHs are sesquiterpenoids that differ by the position and number of epoxidations, while Lepidoptera also make epoxides of homosesquiterpenoids (Goodman and Cusson, 2012, Jindra et al., 2013, Tsang et al., 2020). JH III skipped bisepoxide (JHSB3) appears to be the only JH type in true bugs Heteroptera (Matsumoto et al., 2021), including the kissing bug *Rhodnius prolixus* (Villalobos-Sambucaro et al., 2020), the insect species in which Wigglesworth's performed his pioneering experiments (1934, 1936, 1940, 1948). Ironically, the heteropteran JH was the last determined type (Kotaki et al., 2009).

The biosynthesis of JH begins with Acetyl-CoA via the universal eukaryotic mevalonate pathway and leads to farnesyl pyrophosphate which is then converted (through farnesol and farnesal) to farnesoic acid (FA) (Noriega et al., 2006, Tsang et al., 2020). FA is then methylated and epoxidized, producing JH. Epoxidation and methylation of FA are the final important steps in JH biosynthesis. However, the order of these steps is different in different insects (Fig. 1B, Defelipe et al., 2011, Tsang et al., 2020). In Lepidoptera, FA is first epoxidized and then methylated. In most insects, methylation precedes the epoxidation step. Methyl farnesoate (MF), the non-epoxidized sesquiterpenoid, is the final product in Crustacea, the group of arthropods from which insects evolved. Epoxidation of MF is a novelty in insects (Daimon and Shinoda, 2013) and, as was shown recently, affects reproductive fitness (Nouzova et al., 2021). Nevertheless, MF was detected and seems to play a role in at least some insect species (Wen et al., 2015). The MF epoxidase is encoded by a monophyletic branch of the CYP2 clan P450s unique to insects (Helvig et al., 2004, Dermauw et al., 2020, Nouzova et al., 2021).

Juvenile Hormone Acid Methyl Transferase (JHAMT) is responsible for the methylation step required for the synthesis of JH and MF. A poorly characterized protein called Farnesoic Acid Methyl Transferase (FAMeT or sometimes FAMT), was originally described in Crustacea but its function is unclear, and it may not even be an enzyme. The role of JHAMT in insect JH synthesis has been well documented biochemically (Shinoda and Itoyama, 2003, Minakuchi et al., 2008, Mayoral et al., 2009), using null mutants or gene silencing (Minakuchi et al., 2008, Daimon et al., 2015), and in a recent study addressing the specific structural properties of this enzyme (Guo et al., 2021). As expected, JHAMT appears to be involved in MF synthesis of (at least) some Crustacea (Toyota et al., 2015, Xie et al., 2016).

The lineage-specific idiosyncrasies in the last steps of JH biosynthesis together with the complexity of JH roles in life of insects, invites to thoroughly explore the genetic repertoire that is or might be participating in JH biosynthesis. Given the recent remarkable progress in genome and transcriptome sequencing, we decided to explore available public databases and our in-house genome of the linden bug *P. apterus* for presence of JHAMT, FAMeT, and JH epoxidases. We reconstructed evolution of the three proteins and addressed gene duplication at genomic level in 18 representative species of Arthropoda. We described in more detail the heterogeneity of FAMeT proteins including the conserved alternative splicing of *famet* transcripts. Although our results do not provide experimental evidence for the particular role of the specificities described above, the analysis defines important questions and some hypotheses worth pursuing experimentally.

## 2 Material and methods

### 2.1 Gene discovery

In principle, we used the same approach as in deciphering the evolution of insect insulin receptors and in the overview of the circadian clock setup (Smykal et al., 2020, Kotwica-Rolinska et al., 2022). First, a quick search was performed with the Basic Local Alignment Search Tool (BLAST) using the BLASTP algorithm to identify available annotated JHAMT, FAMeT, and CYP303/305/15 proteins in GenBank. Subsequently, several rounds of order-, family-, and species-based searches were performed in protein (BLASTP algorithm) and transcriptome shotgun assemblies (TSAs; TBLASTN algorithm) using annotated proteins as queries. In selected species (*Drosophila melanogaster*, *Musca domestica*, *Aedes aegypti*, *Hermetia illucens*, *Bombyx mori*, *Manduca sexta*, *Danaus plexippus*, *Tribolium castaneum*, *Apis mellifera*, *Nasonia vitripennis*, *Bemisia tabaci*, *Acyrthosiphon pisum*, *Rhodnius prolixus*, *Nilaparvata lugens*, *Cryptotermes secundus*, *Schistocerca gregaria*, *Daphnia pulex*, and *Centruroides sculpturatus*) we performed search in assembled genomes and mapped identified *jhamt* and *jhamt*-like genes to specific DNA contigs.

Identified proteins were aligned using the MAFFT (Katoh and Standley, 2013) E–INS-i algorithm in Geneious Prime 2022 (Biomatters, Auckland, New Zealand, https://www.geneious.com) and a FAST tree algorithm (Price et al., 2009) in Geneious Prime 2022 (Biomatters, Auckland, New Zealand) was used to infer preliminary trees and identify duplicates. In addition, reciprocal BLAST searches were performed when the identified sequence served as a query in the next BLAST rounds. For outgroups, sequences from representative non-insect arthropods (and for JHAMT even non-arthropods) were retrieved.

### 2.2 Phylogenetic analyses

Sets of identified protein sequences and reference sequences were aligned using the MAFFT algorithm with the E-INS-i algorithm alignment and the BLOSUM80 scoring matrix. Poorly aligned regions were removed, and trees were inferred using RAxML maximum likelihood GAMMA-based model and bootstrap values calculated from 100 replicates. Alternatively, trees were inferred using IQ-TREE maximum likelihood under GAMMA-based model with 1000 ultrafast replicates (Minh et al., 2013).

### 2.3 DNA contigs containing *jhamt* and *jhamt*-like genes

Genes encoding JHAMT and JHAMT-like proteins were searched in RefSeq Representative genomes (refseq_representative_genomes) or RefSeq genome databases (refseq_genomes) in GenBank by using Basic Local Alignment Search Tool (BLAST) with species-specific JHAMT/JHAMT-like protein sequence as a query (TBLASTN). All positive hits, including partial sequences, were manually inspected and putative *jhamt*/*jhamt*-like genes’ open reading frames were collected, translated, and aligned together with known JHAMT/JHAMT-like sequences for reliable *jhamt*/*jhamt-like* identification in Genious Prime 2022 (Biomatters, Auckland, New Zealand). Genes depicted in Fig. 4 and Fig. S2 represent identified *jhamt* (magenta)/*jhamt*-like (yellow) genes with neighboring protein-coding (white arrows) and non-protein-coding (grey arrows) genes. Protein identification number (or designation from alternative protein databases representing the same protein), GenBank gene ID, and contig accession number were presented. Genes flanking *jhamt*/*jhamt*-like genes in Lepidoptera gene synteny Fig. 4C were extracted and protein sequences from *B. mori*, *M. sexta*, and *D. plexippus* prospected for protein signatures with Interproscan plugin (Geneious Prime 2022, Biomatters, Auckland, New Zealand), aligned and unequivocally identified.

### 2.4 *FAMeT* gene models

Models of *famet* genes presented in Fig. 5 were retrieved from GenBank (*D. melanogaster*, *T. castaneum*, *B. mori*, *A. mellifera*), retrieved from GenBank and additional transcripts were mapped (*B. mori*, *C. secundus*), or were built using in-house genomic *P. apterus* assembly (which will be published elsewhere) and Oxford Nanopore Technology (ONT) transcriptomic reads from various tissues. Briefly, *P. apterus* genomic ONT reads were assembled with the assembler Flye version 2.7; Kolmogorov et al., 2019) and the persisting sequencing errors were further polished by the tool Medaka (Oxford Nanopore Technologies) using Illumina genomic reads. The ONT contigs were corrected by mapping Illumina-based reads on ONT reads by using the FM-index Long Read Corrector (FMLRC, version 1.0.0; Wang et al., 2018). The transcript sequence of the *P. apterus famet* gene was searched in an in-house transcriptome assembled from Illumina reads using Basic Local Alignment Search Tool (BLAST) with *D. melanogaster* transcripts as a query. Full-length *famet* transcripts were then searched in ONT cDNA databases, generated from full-length polyA+ mRNAs (from male and female brains, adult female fat body, 4th instar larval epidermis, and embryos) by BLAST, and the hits were mapped to the corrected ONT genomic contig using Minimap2 mapper plugin (version 2.17; Li, 2018) within Geneious Prime 2022 (Biomatters, Auckland, New Zealand) with a default setting. Canonical splice sites were manually located at the contigs according to the mapped exons/transcripts. *P. apterus famet* gene model was submitted to GenBank under accession number OP737817. Transcripts representing 1x pfam12248–DUF3421 and/or 2x pfam12248–DUF3421 were acquired from GenBank and mapped to the corresponding gene model: LOC101739845 of *B. mori* and LOC111863761 of *C. secundus*.

## 3 Results

### 3.1 Functional Juvenile hormone acid Methyl transferases (JHAMTs)

**Fig. 2:**
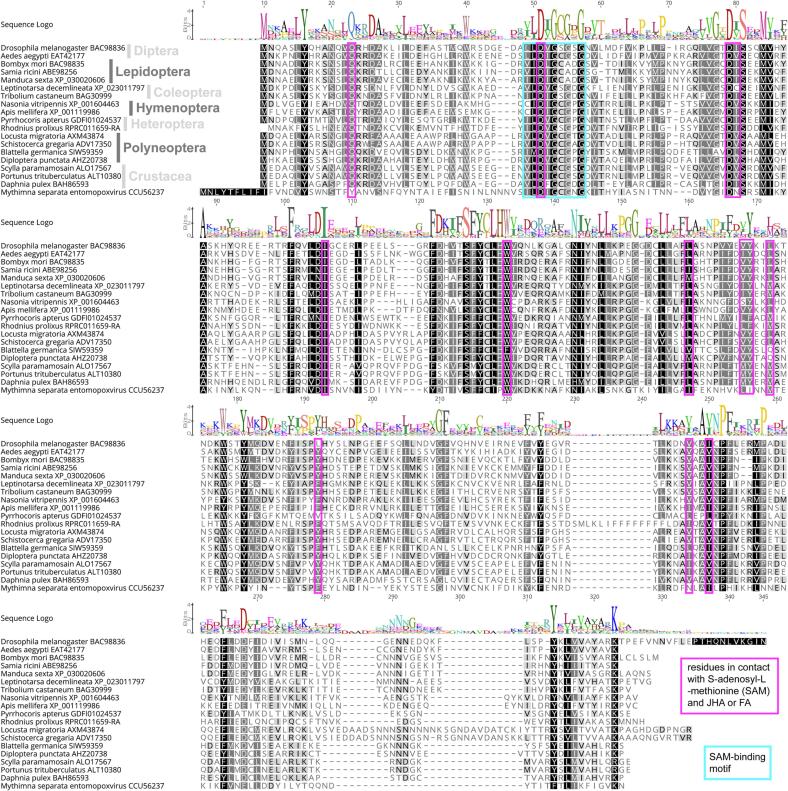
Protein alignment of functionally tested JHAMTs. JHA-methyltransferase activity was confirmed either biochemically *in vitro* using recombinant proteins, by gene silencing that reduced JH titers, or by strong expression in the *corpus allatum*: *Drosophila melanogaster* (CG17330 = BAC98836; Shinoda and Itoyama, 2003, Niwa et al., 2008), *Aedes aegypti* (EAT42177; Mayoral et al., 2009), *Bombyx mori* (BAC98835; Shinoda and Itoyama, 2003, Daimon et al., 2015), *Manduca sexta* (XP_030020606, Yin et al., 2020), *Samia ricini* (ABE98256; Sheng et al., 2008); *Tribolium castaneum* (BAG30999.1; Minakuchi et al., 2008), *Leptinotarsa decemlineata* (XP_023011797; Fu et al., 2015), *Apis mellifera* (XP_001119986; Li et al., 2013), *Nasonia vitripennis* (XP_001604463; Mukai et al., 2022), *Rhodnius prolixus* (RPRC011659-RA, Villalobos-Sambucaro et al., 2020), *Pyrrhocoris apterus* (GDFI01024537, Hejnikova et al., 2022), *Locusta migratoria* (AXM43874; Guo et al., 2020), *Schistocerca gregaria* (ADV17350; Marchal et al., 2011), *Blattella germanica* (SIW59359; Dominguez and Maestro, 2018), *Diploptera punctata* (AHZ20738; Huang et al., 2015), *Daphnia pulex* (BAH86593; Toyota et al., 2015), the mud crab *Scylla paramamosain* (ALO17567; Zhao et al., 2022), the swimming crab *Portunus trituberculatus* (ALT10380; Xie et al., 2016), and a virus *Mythimna separata entomopoxvirus* (MySEV; CCU56237; Takatsuka et al., 2017). The cyan box indicates the conserved S-adenosyl-l-methionine (SAM) binding motif and the magenta boxes highlight the residues in contact with SAM and substrates.

First, we aligned protein sequences of all JHAMTs with experimentally confirmed function, that is their activities were confirmed either biochemically *in vitro* using recombinant proteins, by gene silencing that reduced JH titers, or by strong expression in the *corpus allatum*. The dataset included representatives of all major insect orders, Crustacea, and even a viral sequence that was probably hijacked from a bacterial genome (Fig. 2). Although the alignment highlighted conserved regions, such as the S-adenosyl-l-methionine (SAM)-binding motif, the sequences were surprisingly diverse even among related species, e.g., within the Polyneoptera (represented by two locust and two cockroach species). Therefore, a thorough phylogenetic analysis of JHAMT and JHAMT-like proteins in animals was required.

### 3.2 JHAMT history in insects

First, we systematically explored the GenBank for JHAMT-like proteins and JHAMT-like coding transcripts in all major insect orders but also included Collembola, Crustacea, Chelicerata, and representative deuterostomian species (sea urchin, lancelet *Branchiostoma*). Furthermore, in 18 selected species (16 insects, crustacean *Daphnia pulex* and scorpion *Centruroides sculpturatus*) we prospected assembled genomes and identified *jhamt* and *jhamt*-like genes (we prefer to use the term *jhamt*/JHAMT only for genes/PROTEINS whose role in JH biosynthesis was supported biochemically, by dsRNA-mediated RNA interference (RNAi), or by expression analysis. The remaining sequences are considered JHAMT-like even if there is just one JHAMT-like identified in a particular species). The subsequent phylogenetic analyses, performed on predicted protein sequences, had three objectives: (*i*) Is the JHAMT phylogeny consistent with the relationships of the organisms? (*ii*) In which species and insect lineages do we find *jhamt*/*jhamt*-like multiplications? (*iii*) Are these multiplications independent, i.e., species- or genus-specific?

**Fig. 3:**
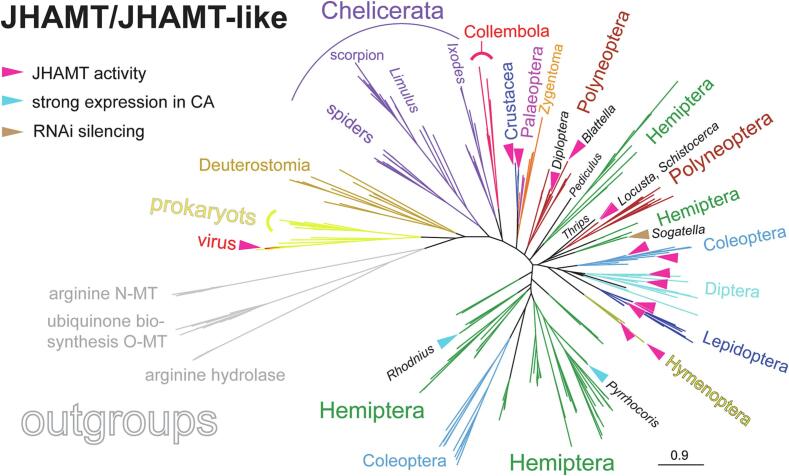
Phylogeny of JHAMT, JHAMT-like, and related methyltransferases. The tree was inferred from protein alignment using the LG + F + G4 model in IQ-TREE with 1000 ultrafast bootstrap replicates. The major taxonomic groups are color coded. See Supplementary Fig. 1 for the detailed tree, bootstrap values, and all accession numbers. The magenta triangles point to proteins for which JHA–methyltransferase (JHAMT) activity was confirmed either biochemically *in vitro* or by gene silencing that reduced JH titers. The turquoise triangle indicates JHAMT, which is highly expressed in the *corpus allatum* of the linden bug *Pyrrhocoris apterus* and *Rhodnius prolixus*, and the brown triangle indicates JHAMT in the white-backed planthopper *Sogatella furcifera*, where the RNAi-suppressed ovarian development was rescued by JH–mimic application.

**Fig. 4:**
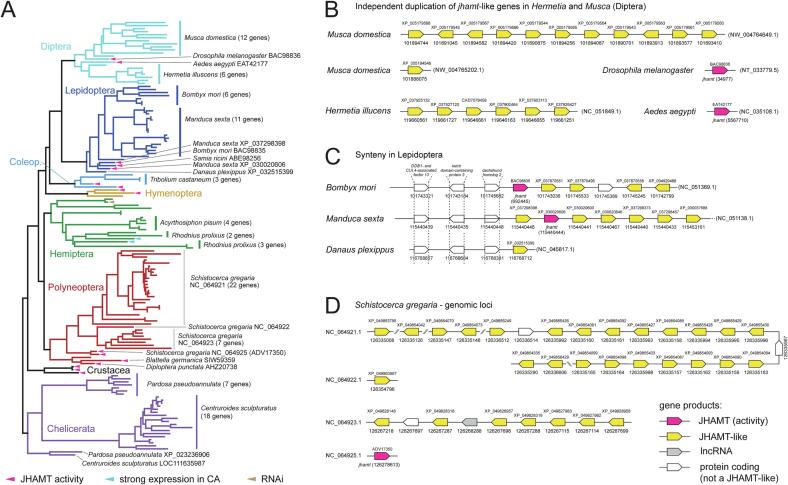
Genomic analysis of *jhamt* and *jhamt*-like genes in selected representative arthropod species. (A) Phylogenetic tree inferred from established JHAMTs and protein sequences identified in 18 arthropod genomes (LG + F + G4 model in IQ-TREE with 1000 ultrafast bootstrap replicates). (B) Genomic organization of *jhamt-*like genes in four dipteran species. (C) Comparison on lepidopteran *jhamt-*like paralogs points to a clear synteny upstream of *jhamt* genes, whereas the region downstream evolved rapidly. (D) Organization of 31 *jhamt*-like genes in four genomic loci in *Schistocerca gregaria*.

The protein alignment contains positions that are either highly conserved or highly variable even within the JHAMT proteins. Therefore, identifying an appropriate outgroup is not trivial. Even though some studies have compared FAMeT with JHAMT (Hui et al., 2010), we found the sequence differences between these two protein groups too large for (any) phylogenetic analyses. Therefore, we identified three groups of sequences closely related to JHAMT: Arginine methyl transferases, arginine hydrolases, and ubiquinone O-methyl transferases (ubiquinone biosynthesis O-MT). The phylogenetic tree depicted in Fig. 3 shows the phylogeny of JHAMT and JHAMT-like proteins from arthropods compared to homologous sequences identified in basal Deuterostomia. Furthermore, this analysis includes prokaryotic sequences and sequences obtained from viruses (see the full tree with bootstrap supports and accession numbers in Supplementary Fig. S1). In a parallel analysis, genomes of 18 arthropod species were prospected for *jhamt* and *jhamt-like* genes, their mutual position and orientation on DNA contigs were depicted (Fig. S2), and the phylogeny of putative protein sequences was determined (Fig. 4).

JHAMT and JHAMT-like sequences formed several clusters: The first cluster contains prokaryotic sequences and sequences obtained from viruses (Fig. 3). One of them, *Mythima separata entomopoxvirus* CCU56237, proved to be a potent JHAMT in lepidopteran hosts (Takatsuka et al., 2017). Sequences homologous to JHAMT have even been identified in basal Deuterostomia such as the lancelet *Branchiostoma belcheri*, the acorn worm *Saccoglossus kowalevskii* (Hemichordata), and in several species of Echinodermata (*Anneissia japonica*, *Lytechinus variegatus*, *Strongylocentrotus purpuratus*), but no reasonable homologs have been found in vertebrates.

The second cluster contained sequences of Chelicerata, which were assembled into three monophyletic groups of spider (*Nephila pilipes*, *Pardosa pseudoannulata*), scorpion (*Centruroides sculpturatus*), and horseshoe crab (*Limulus polyphemus*) sequences. Another set of chelicerate sequences (tick, spiders, scorpion, and horseshoe crab) forms a sister group to JHAMT-like sequences of Collembola, Crustacea, and Insecta.

The analysis does not allow to define a clear ancestral sequence to the insect JHAMTs. At least, it is safe to say that Chelicerata, Collembola, and Crustacea branch at the base of a large group containing insect sequences. Branching within this group is sometimes poorly supported, but some conclusions can be drawn. Holometabolan species are grouped into two branches: one containing Hymenoptera, Lepidoptera, Diptera, Siphonaptera, and the majority of coleopteran sequences. This group contains all holometabolan JHAMT proteins whose enzymatic ability to methylate JH acid has been experimentally confirmed. The holometabolan sequences outside this monophyletic group are all from the dung beetle *Onthophagus taurus*. Polyneoptera, a large assemblage of several orders, was divided into two groups. One contains only Orthoptera represented by various species of locusts and includes two established JHAMT proteins (*Locusta migratoria*, *Schistocerca gregaria*). The remaining polyneopteran sequences from the orders Orthoptera, Plecoptera, Blattodea (including termites), and Phasmida were grouped together. Two experimentally tested JHAMTs (*Blattella germanica* and *Diploptera punctata*) are located here. The last major insect group analyzed, Hemiptera, splits into several groups. JHAMT has been experimentally tested in at least three species of Hemiptera: the white-backed planthopper *Sogatella furcifera*, the linden bug *Pyrrhocoris apterus*, and the kissing bug *Rhodnius prolixus*. Each of these JHAMTs is located in a different part of the tree.

### 3.3 Genomic organization and duplication of *jhamt* and *jhamt*-like genes

The number of identified *jhamt*/*jhamt*-like sequences varies considerably among species. In some insects, only a single JHAMT(-like) has been identified, e.g., in *Drosophila melanogaster*, *Blattella germanica*, *Cryptotermes secundus*, *Nilaparvata lugens*, *Apis mellifera*. Not only do these species represent different insect orders, but their related species often possess more than one JHAMT (see Supplementary Figure S1 for the full tree).

To investigate gene duplication(s), the genomes of 18 representative arthropod species were examined for *jhamt*/*jhamt*-like genes. While *Drosophila melanogaster* (Diptera), *Aedes aegypti* (Diptera), *Danaus plexippus* (Lepidoptera), *Apis mellifera* (Hymenoptera), *Nilaparvata lugens* (Hemiptera), *Cryptotermes secundus* (Polyneoptera), and *Daphnia pulex* (Crustacea) contain only one *jhamt*(-like) gene in their genomes, as many as 31 genes were identified in *Schistocerca gregaria* (Fig. S2). Phylogenetic analysis revealed several species/genus-specific gene multiplications: 12 gene copies of *Musca domestica* form one monophyletic group branching next to *D. melanogaster*, whereas six copies of *Hermetia illucens* form a separate monophyletic group with mosquito JHAMT at its base (Fig. 4A). Consistent with phylogenetic analysis, the organization of *jhamt/jhamt-like* copies on DNA contigs differs between *Musca* and *Hermetia* (Fig. 4B).

In Lepidoptera, only one JHAMT-like protein identified in *D. plexippus* branches together with three established JHAMTs: BAC98835 from *Bombyx*, ABE98256 from *Samia*, and XP_030020606 from *Manduca*. However, additional 6 JHAMT-like coding genes were identified in *Bombyx* and 11 in *Manduca*; all of them form a monophyletic group next to the established lepidopteran JHAMTs. Within this monophyletic group, *Bombyx* and *Manduca* form separate subgroups (Fig. S2). Analysis of the lepidopteran loci revealed a clear synteny among *jhamt* loci with conserved upstream region, whereas *jhamt*-like multiplication in *Manduca* and *Bombyx* continued in only one direction of the contigs (Fig. 4C). Notably, the orientation of *jhamt*-like copies is different between *Manduca* and *Bombyx*, which, in conjunction with the topology of the tree, suggests independent gene duplications and/or additional chromosomal rearrangements.

A remarkable *jhamt*-like multiplication in *Schistocerca* is localized to four genomic contigs. Two of these contain multiple *jhamt*-like copies (22 and 7, respectively; Fig. 4D). Phylogenetic reconstruction of the predicted JHAMT-like proteins reveals that proteins encoded from the same contig branch together (Fig. 4D and Fig. S2).

Phylogenetic analysis identified several additional species-specific duplications, including a monophyletic group of four copies in *Acyrthosiphon pisum* (Hemiptera), three and two copies in *Rhodnius prolixus* (Hemiptera), seven gene copies in *Pardosa pseudoannulata* (spider, Chelicerata), and a monophyletic group of 18 genes in *Centruroides sculpturatus* (scorpion, Chelicerata). The transcriptomic data suggest additional lineage-specific gene duplications, including common duplications, such as duplications shared between *Largus californicus* and *Pyrrhocoris apterus* (Heteroptera, Pyrrhocoroideae), or between *Tenebrio molitor* and *Tribolium castaneum* (Coleoptera, Tenebrionidae). But in general, gene copy number changes rapidly even between species.

### 3.4 FAMeT evolution

**Fig. 5:**
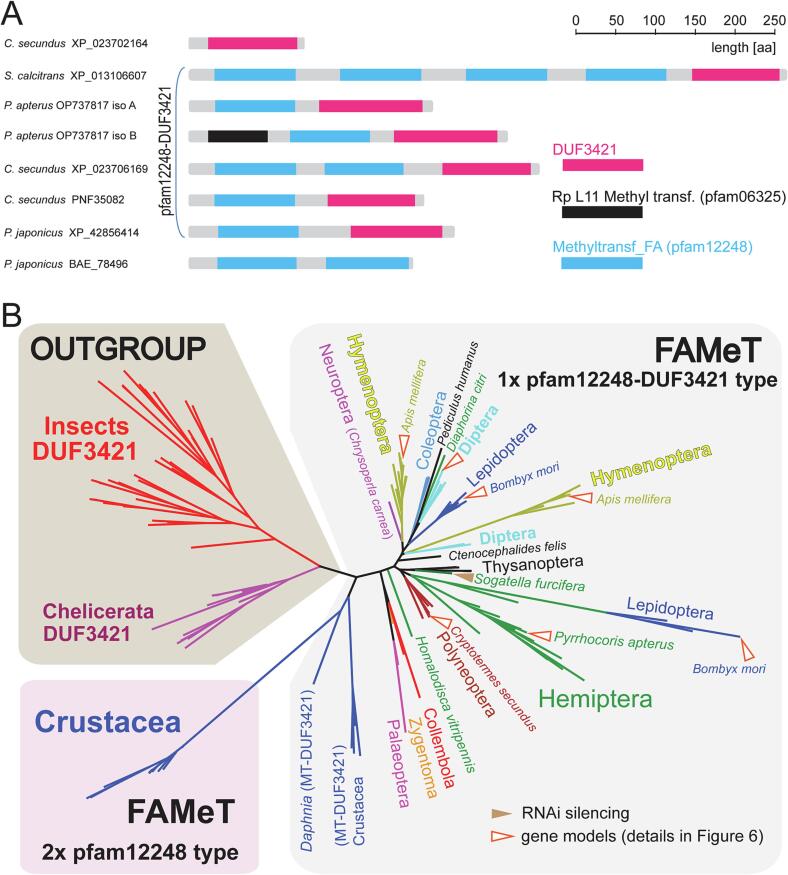
Domain structure and phylogeny of FAMeT proteins. (A) Two FAMeT types are detected in the majority of Crustacea (represented by *Penaeus japonicus*). Two pfam12248 (methyltransf_FA) domains are either arranged in tandem (type 2x pfam12248), or pfam12248 is located upstream of the DUF3421 domain (type pfam12248–DUF3421). In insects and *Daphnia*, only the pfam12248–DUF3421 type was identified. In insects, one or two MT domains (represented here by termite *Cryptotermes secundus*) are usually found in FAMeT proteins as a result of alternative splicing of mRNA expressed from the same gene (see Fig. 6). However, in Muscomorpha, up to four pfam12248 domains can be found as exemplified by *Stomoxys calcitrans*. In the linden bug *Pyrrhocoris apterus*, only one pfam12248 domain is found. However, alternative transcription start sites and splicing result in a rare protein variant with an additional upstream Rp L11 methyltransferase domain (black; also known as pfam06325). The DUF3421 domain (magenta) is also present alone in natterin–3–like proteins (represented by *C. secundus* XP_023702164). (B) Evolutionary reconstruction of the FAMeT proteins. The tree was inferred from the protein alignment using the LG + F + G4 model in IQ-TREE with 1000 ultrafast bootstrap replicates. The major taxonomic groups are color-coded. DUF3421/natterin-3-like proteins were used as outgroups. The pfam12248–DUF3421 type of FAMeT is found in all insects and all crustacean species examined. The 2x pfam12248 type is only found in most crustacean species, with the exception of *Daphnia* spp. where only the pfam12248–DUF3421 was identified. See Fig. S3 for the detailed tree, bootstrap values, and accession numbers.

Given the proposed role of FAMeT in the final steps of JH synthesis, we systematically investigated the evolution of FAMeT proteins in arthropods. Proteins containing a pfam12248 domain (called methyltransf_FA by pfam curators) were identified only in Crustacea and in insects, but not in the other arthropods, although we specifically searched for them using different crustacean or insect sequences as a query. Interestingly, the FAMeT proteins differ in the combination of domains. In most Crustacea, two FAMeT types were identified (Fig. 5A). In the first type (2x pfam12248), the protein consists of two tandem domains separated by a linker approximately 40 amino acids (aa) long. In the second type (pfam12248-DUF3421), the FAMeT contains pfam12248 domain upstream of DUF3421, a domain of an unknown function that is similar in length to the pfam12248 domain (Figure S2). In insects, only the pfam12248-DUF3421 type is present, but a detailed examination revealed additional complexity in a number of pfam12248 domains. Most insect FAMeT proteins contain one or two pfam12248 domains (1-2x pfam12248-DUF3421), but up to as many as four pfam12248 domains have been identified in some Muscomorpha (Fig. 5A).

**Fig. 6:**
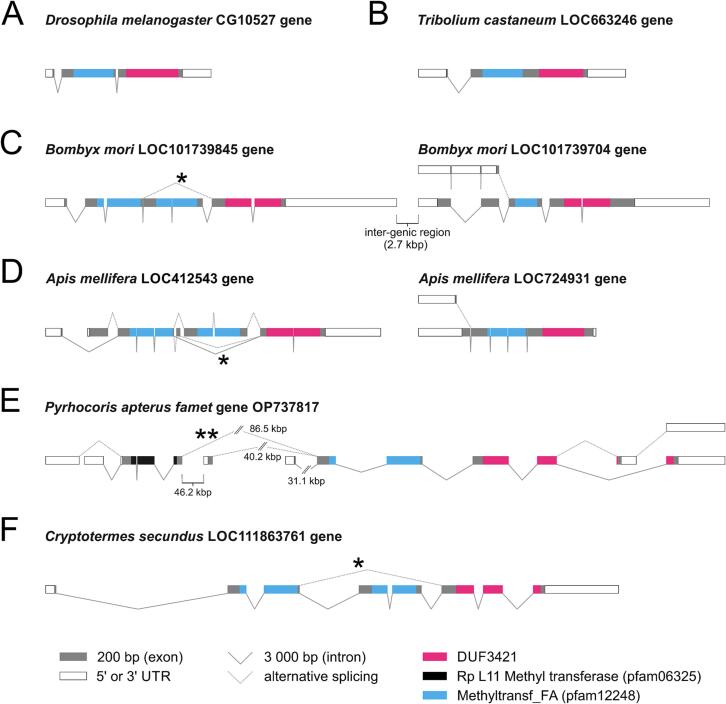
Alternative splicing is a conserved mechanism producing 1x pfam12248 and 2x pfam12248 variants of FAMeT in most insects. The compact *famet* genes (A) of the fruit fly *Drosophila melanogaster* and (B) of the red flour beetle *Tribolium castaneum* encode only one pfam12248 copy (blue). (C) Two paralogous *Bombyx mori* genes are arranged in tandem in the genome; the asterisk indicates alternative splicing of the LOC101739845 gene resulting in transcripts encoding versions with one or two pfam12248 copies, respectively. In LOC101739704, alternative splicing influences the N part of the predicted proteins, however, the only pfam12248 domain is located downstream. (D) A similar situation is observed in the honeybee *Apis mellifera*, where alternative splicing of LOC412543 results in multiple isoforms including versions with only one pfam12248 domain (indicated by asterisks). (E) In the linden bug *Pyrrhocoris apterus*, one gene (OP737817) encodes the common version with one pfam12248 domain upstream of DUF3421. Alternative transcription start sites and splicing (indicated by two asterisks) result in a protein with an additional upstream Rp L11 methyltransferase domain (pfam06325). (F) In termite *Cryptotermes secundus*, only one *famet* gene has been identified, again, with the capacity to encode two- and one– pfam12248 variants (asterisk) of FAMeT. The gene models presented here were reconstructed by mapping available mRNA transcripts to genomic loci.

In rare cases, such as in the linden bug *P. apterus*, we have identified a protein with an additional Rp L11 methyltransferase domain (pfam06325), an AdoMet-dependent transferase domain, located upstream of 1-2x pfam12248-DUF3421. The mRNA sequences were confirmed by multiple reads obtained from Oxford Nanopore Technology sequencing (direct sequencing of full-length cDNA molecules), therefore, do not reflect an artifact obtained *in silico* during the assembly. Furthermore, Rp L11 methyltransferase (pfam06325) encoding exons map onto one genomic locus upstream of 1x pfam12248-DUF3421 (Fig. 6E).

Therefore, we performed a phylogenetic reconstruction of the 2x pfam12248, pfam12248-DUF3421, and (only–) DUF3421-containing domain proteins from species representing major arthropod lineages. The maximum likelihood tree revealed a clear division of all analyzed sequences into four clusters: the insect DUF3421 cluster, the chelicerate DUF3421 cluster, the crustacean FAMeT cluster which can be further divided into the 2x pfam12248 type and the pfam12248-DUF3421 type, and the insect FAMeT clusters containing only pfam12248-DUF3421 type proteins (Fig. 5B and Fig. S4). Phylogenetic analysis and detailed sequence comparison revealed gene duplication of FAMeT in Lepidoptera, Hymenoptera, and Thysanoptera. The remaining pfam12248-DUF3421 type protein sequences were identical within species, but versions with either one (1x pfam12248-DUF3421) or two pfam12248 domains (2x pfam12248-DUF3421) were identified in the majority of insects. The perfectly conserved sequences implied alternative splicing as the most likely source of this diversity (see Fig. 6).

### 3.5 FAMeT gene structure

To clarify the origin of 1x pfam12248-DUF3421 and 2x pfam12248-DUF3421 variants, we analyzed the gene models in selected insect representatives (Fig. 6). Not only were the gene models analyzed at the DNA level, but we also mapped the available transcripts to reliably identify alternative transcripts. In the compact genes of *Drosophila* and *Tribolium*, only the 1x pfam12248-DUF3421 is encoded (Fig. 6A, B). In *Bombyx* and *Apis*, duplication resulted in two *famet* genes. Whether both genes originated from a single duplication event, or whether the genes were duplicated separately in Hymenoptera and Lepidoptera, remains unclear. The first hypothesis is slightly favored by similarities in the gene products. In both species, one of the genes encodes only the 1x pfam12248-DUF3421 variant, whereas the other gene encodes both the 2x pfam12248-DUF3421 and 1x pfam12248-DUF3421 versions, the latter resulting from alternative splicing (Fig. 6C, D). Similar alternative splicing resulting in 1x pfam12248-DUF3421 and 2x pfam12248-DUF3421 transcripts was also found in the drywood termite *C. secundus* (Fig. 6F). In the linden bug *P. apterus,* the 1x pfam12248-DUF3421 version is encoded by one *famet* gene. Interestingly, expression from two upstream alternative transcriptional start sites in *P. apterus* results in an N-terminal extension of the protein with the Rp L11 methyltransferase domain (pfam06325), which is an AdoMet-type of methyl transferase (Fig. 6E).

### 3.6 CYP15 – Insect JH epoxidase

The evolution of JH epoxidase was thoroughly addressed in a functional study by Nouzova et al. (2021), who not only elegantly proved the role of *Aedes* CYP15 in the epoxidation of JH, but also reconstructed the evolution of CYP15 and closely related proteins in insects (see the supplementary material of that study). Therefore, we had to identify for our analysis only CYP15, CYP303, and CYP305 from a few additional species relevant to the comparison presented here. Essentially, we obtained trees with comparable branching to Nouzova et al. (2021), in which no CYP15 was identified outside Insecta (see supplementary Fig. S6 for details and accession numbers). All insect species studied contained only one CYP15 gene, although hypothetical alternative splice variants and assembly artifacts were occasionally identified. Consistent with previous studies, we did not identify CYP15 in cyclorrhaphan Diptera, such as *Drosophila*, *Stomoxys* and *Musca*, whereas *Hermetia* and mosquitoes contain CYP15.

## 4 Discussion

**Fig. 7:**
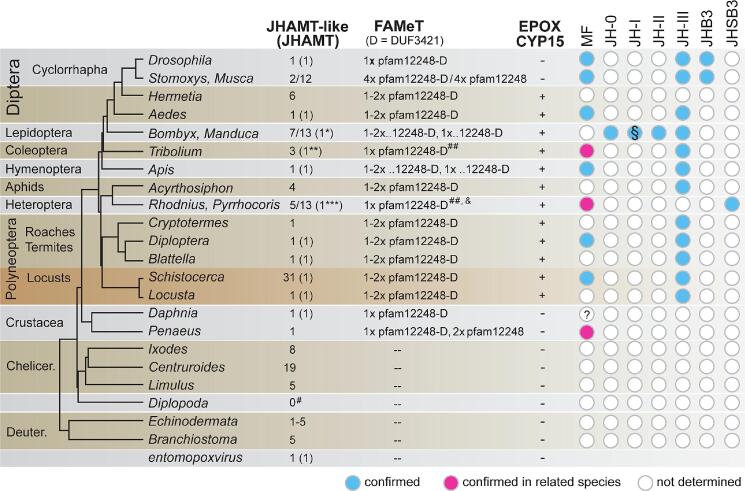
A summary of the major genetic changes in the (putative) enzymes involved in the final steps of the synthesis of Methyl Farnesoate (MF) and various forms of Juvenile Hormone (JH). The tree on the left shows the current view of phylogenetic relationships among the depicted species (Misof et al., 2014). The first column indicates the number of identified JHAMT and JHAMT-like protein-encoding genes for each species, while the value in parentheses indicates the proteins for which biological activity in MF/JH synthesis has been confirmed. *although two *B. mori* JHAMTs are functional methyltransferases *in vitro* (Guo et al., 2021), knockout of a single JHAMT (BAC98835) is sufficient to show higher embryonic mortality and precocious metamorphosis *in vivo* (Daimon et al., 2015); **only one protein from *T. castaneum* is capable of JHA methylation, whereas the other two proteins were inactive on JHA; *** up to 13 clearly different transcripts were identified in *P. apterus*, but only one of them is strongly expressed in the *corpus allatum*; ^#^ the loss of JHAMT is well-documented for Diplopoda using genome data from five species (So et al., 2022). The FAMeT proteins belong to the 2x pfam12248 type and the pfam12248–DUF3421 type (pfam12248–D). The latter contains either only one pfam12248 domain (1x pfam12248–D), four domains in *Stomoxys calcitrans* (4x pfam12248–D), but most often the alternative splicing of the *famet* mRNA results in both 1x pfam12248 and 2x pfam12248 versions (1–2x pfam12248–D). ^##^ in Heteroptera and Coleoptera, only FAMeTs with one pfam12248 upstream of DUF3421 were found. ^&^ In the linden bug *Pyrrhocoris apterus*, a rare variant with an additional methylase domain was identified. However, this upstream domain belongs to Rp L11 methyltransferase domain (pfam06325), not pfam12248 domain. Cytochrome P450 CYP15 is a genetic novelty in insects responsible for JH epoxidation and has been lost secondarily in cyclorrhaphan Diptera, such as *Drosophila*, *Stomoxys*, and *Musca*. Circles indicate detected JH types and MF are shown for each species (for details and references, see supplementary material for Fig. 7). Question mark (?) in MF for *Daphnia* refers to the discrepancy: although MF has not been directly detected in this species (Toyota et al., 2021), MF is considered as an innate JH of all Crustacea. § indicate JH-I and 4-Methyl JH-I detected in *Manduca sexta.*

In this study, we investigated the evolution of proteins involved in the final steps of biosynthesis of MF and JH (Fig. 7). We would like to highlight some remarkable trends that we observed and summarize some implications and suggestions that emerge from this and other studies. First, the *jhamt* gene tends to replicate in many species. Numerous paralogs have often arisen independently, and this can be well documented in some phylogenetic trees (Fig. 4A, S1, S3). Often, paralogs are located within the same genomic locus, which is well illustrated in *Musca*, *Hermetia*, *Tribolium*, *Manduca*, *Bombyx*, *Schistocerca*, *Pardosa,* and *Centruroides* (Fig. 4B, C, D, S2; Minakuchi et al., 2008, Yang et al., 2021). Furthermore, paralogs located on the same DNA contig tend to branch together in phylogenetic analyses (Fig. 4A), which implies that they originated from the same ancestor independently in each arthropod lineage. It would be interesting to investigate the mechanism behind these duplications and whether they share a common general mechanism for all species.

It should be noted that mere multiplication does not mean that all resulting genes encode functional JHAMT proteins, even though the original gene might have been involved in JH biosynthesis and the paralogs include conserved S-adenosyl-l-methionine (SAM) binding motif and the residues facilitating contact with SAM and substrates. Careful biochemical evaluation of three *T. castaneum* paralogs revealed that only one of them is the functional JHAMT, while the other two have an unknown function in this beetle (Minakuchi et al., 2008). Therefore, the ability of a JHAMT candidate to participate in JH biosynthesis should be further verified by examining the biological activity of the recombinant protein and ideally by genetic knockdown leading to low JH titers. At the same time, understanding the biochemical function of JHAMT-like homologs that do not participate in JH biosynthesis will help to elucidate the selection pressures on the evolution of this gene and, to some extent, estimate the likelihood of recruiting methylase to participate in JH biosynthesis. It will be interesting to see whether all insect functional JHAMTs evolved only once and retained their role in MF/JH biosynthesis, or is it possible that some JHAMTs regained this ability when *jhamt*-like was mutated? The presence of multiple paralogs in some insects provides a good opportunity for the latter. Finally, is it possible that some insect species have more than one JHAMT involved in MF/JH biosynthesis?

A notable clue in narrowing down the possible candidates is a strong expression in the JH-producing tissue, the *corpus allatum*. This approach led to the successful identification of JHAMT in the kissing bug *R. prolixus* (Villalobos-Sambucaro et al., 2020), although purely computational prediction led to the misidentification of a JHAMT-like protein that is not even expressed in CAs (Mesquita et al., 2015). Although CA seems to be the primary place of JHAMT expression and function, a high number of *jhamt*/*jhamt*-like genes in some insect groups provokes a ‘heretic’ possibility of JHAMTs to be expressed and function in other tissue(s) than in CA. Whether *R. prolixus jhamt*-like gene identified by Mesquita et al. (2015) is expressed in non-CA tissue is not known but *P. apterus jhamt* ‘MAG-type’ gene (GDFI01048469) is expressed preferentially in male accessory glands (MAG), where expression of canonical CA-type *jhamt* (GDFI01024537) is low (Hejnikova et al., 2022). Whether MAG can be a place of JH synthesis is unclear, even though *Aedes aegypti* (Clifton et al., 2014) and *Hyalophora cecropia* (Lepidoptera) (Shirk et al., 1976) MAGs contain JH. Furthermore, the *Hyalophora* enzyme is a very specific JHAMT producing JH I from JH I acid (Peter et al., 1979). Unfortunately, no genome of *Hyalophora* species is available. Given the *jhamt*/*jhamt*-like multiplication in *Bombyx* and *Manduca*, one can expect multiple *jhamt*/*jhamt*-like paralogs in *H. cecropia*. Indeed, our preliminary inspection of the non-annotated genome draft of *Samia ricini*, a saturnid species belonging to the same tribe as *Hyalophora* (Attacini), revealed two contigs with *jhamt*-like hits: Sr_HGAP_JL_scaf_3 (BLXV01000003.1) and Sr_HGAP_JL_scaf_5 (BLXV01000005.1). The latter one contains multiple hits, thus, once the genome is well annotated or a good transcriptome is available, one can define the actual number of *jhamt*-like copies in this saturnid species.

The presence of multiple *jhamt*/*jhamt*-like copies in individual species brings us to a technical issue that is important for determining which of these paralogs are responsible for the biosynthesis of MF and JH. RNAi is a powerful method for functional analysis in many insect species. Usually, dsRNA fragments of several hundred base pairs (bp) long are produced *in vitro* and injected into the experimental animal, where DICER cleaves the dsRNA into 21–24 bp siRNAs, which then bind the mRNA and lead to its specific degradation. Therefore, mRNAs with identity or high similarity spanning even only 21–24 nucleotides can be silenced simultaneously. This drawback of RNAi technology is relatively easily remedied when silencing single-copy genes: Parallel experiments are performed with non-overlapping dsRNA fragments so that the risk of identical off-targeting is minimal. However, the situation is quite different in the case of multiple and highly similar sequences such as *jhamt*-like paralogs. Therefore, in addition to knocking down expression of the target sequence, additional paralogs sharing sequence similarity might be silenced as well. This technical aspect of RNAi should be kept in mind when silencing *jhamt* and interpreting the outcome.

The ability to catalyze the methylation of JH III acid can even be achieved by an enzyme used in prokaryotes and hijacked by a virus, as was elegantly demonstrated by Takatsuka et al. (2017). In this case, a mere *in silico* sequence analysis would not predict such a dramatic phenotype observed in the metamorphosis of larvae infected by *Mythimna separata entomopoxvirus* (MySEV). It will be interesting to see if additional viruses have independently hijacked methyltransferase genes from different prokaryotic hosts. The recruited JHAMT in entomopoxviruses is still related to insect JHAMT, albeit distantly (see Fig. 2. and Takatsuka et al., 2017). Is it possible to harness a rather different type of methyltransferase for this task? If so, has this already happened in nature? This brings us to the second gene studied here *in silico* - *famet*.

FAMeT has been proposed as an alternative methylase to JHAMT, however, its capacity to methylate FA or JHA has never been reliably shown *in vitro*. Moreover, mutagenesis of FAMeT in the fruit fly *Drosophila melanogaster* had no effect on the levels of MF or JH (Burtenshaw et al., 2008).

Our phylogenetic analyses point to several aspects of FAMeT proteins. Firstly, FAMeT proteins can be divided into distinct types: 2x pfam12248 type identified only in the majority of Crustacea, and pfam12248 in combination with DUF3421, which is found in *Daphnia* and insects. The latter type can contain either one or two pfam12248 domains, which is often the result of alternative splicing. Secondly, transcriptomic analyses of *P. apterus* identified FAMeT variant with an additional upstream Rp L11 methyltransferase domain (pfam06325). Although we do not want to propose that this AdoMet-dependent transferase domain is capable of FA or JHA methylation, the fusion of FAMeT with additional domains should be kept in mind when interpreting (and designing) RNAi experiments. It will be interesting to see if a similar fusion exists in the white-backed planthopper *Sogatella furcifera*, a hemipteran insect in which *famet* silencing affected reproduction (Zhou et al., 2021).

The final key step required to produce a potent JH is epoxidation. The key enzyme CYP15, originally identified in the cockroach *Diploptera punctata* (Helvig et al., 2004), is a novelty among insects (Nouzova et al., 2021). Unlike commonly duplicated *jhamt* genes, *cyp15* does not appear to be duplicated in any insect species (Nouzova et al., 2021), which contrasts to several species- and group-specific expansions of other *cyp* genes in some arthropods (Dermauw et al., 2020). Interestingly, *cyp15* is lost in cyclorrhaphan flies. Therefore, a different epoxidase must have been recruited in this subgroup of Diptera (Wen et al., 2015). Whether a similar epoxidase is also involved in insects with functional CYP15 remains unresolved. The JH of Heteroptera, so-called juvenile hormone III skipped bisepoxide (JHSB3), contains a second epoxide group. Neither the mechanism of synthesis nor the possible involvement of an additional epoxidase is known.

Our study started as a small bioinformatic exercise aimed at explaining why multiple *jhamt* genes exist in the linden bug *P. apterus* (Hejnikova et al., 2022). Apparently, the evolution of the JHAMT and FAMeT proteins is more complex than originally appreciated and even the relatively quick bioinformatic analyses provided more questions than answers. Answering some of these questions could be quite a challenge, an enjoyable challenge. Perhaps this trend is also in line with the legacy of beloved Prof. Frantisek Sehnal.

## Author Contributions

DD and VS designed the study and wrote the manuscript. DD prospected GenBank and performed phylogenetic analyses. VS established the gene models.

## Declaration of Competing Interest

The authors declare that they have no known competing financial interests or personal relationships that could have appeared to influence the work reported in this paper.

## Data Availability

Data will be made available on request.
