# Supplementary figures and images for: Evolution of proteins involved in the final steps of juvenile hormone synthesis
Source: J Insect Physiol. 2023 Mar;145:104487. doi: 10.1016/j.jinsphys.2023.104487 (PMC10015273; doi:10.1016/j.jinsphys.2023.104487)

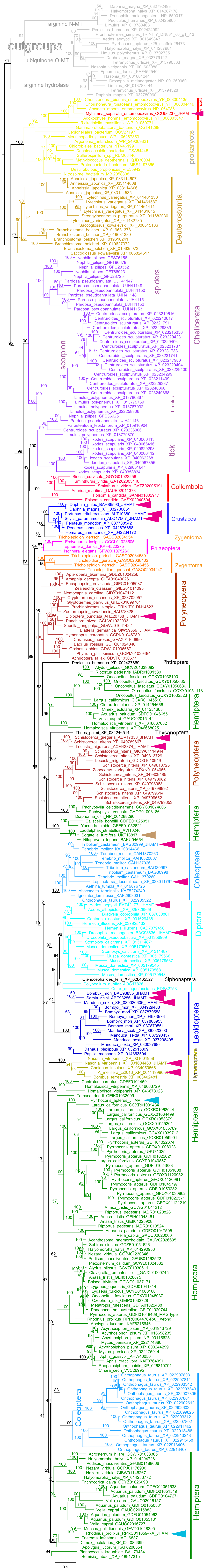

Supplement: Supplementary data 1 [file mmc1.pdf]

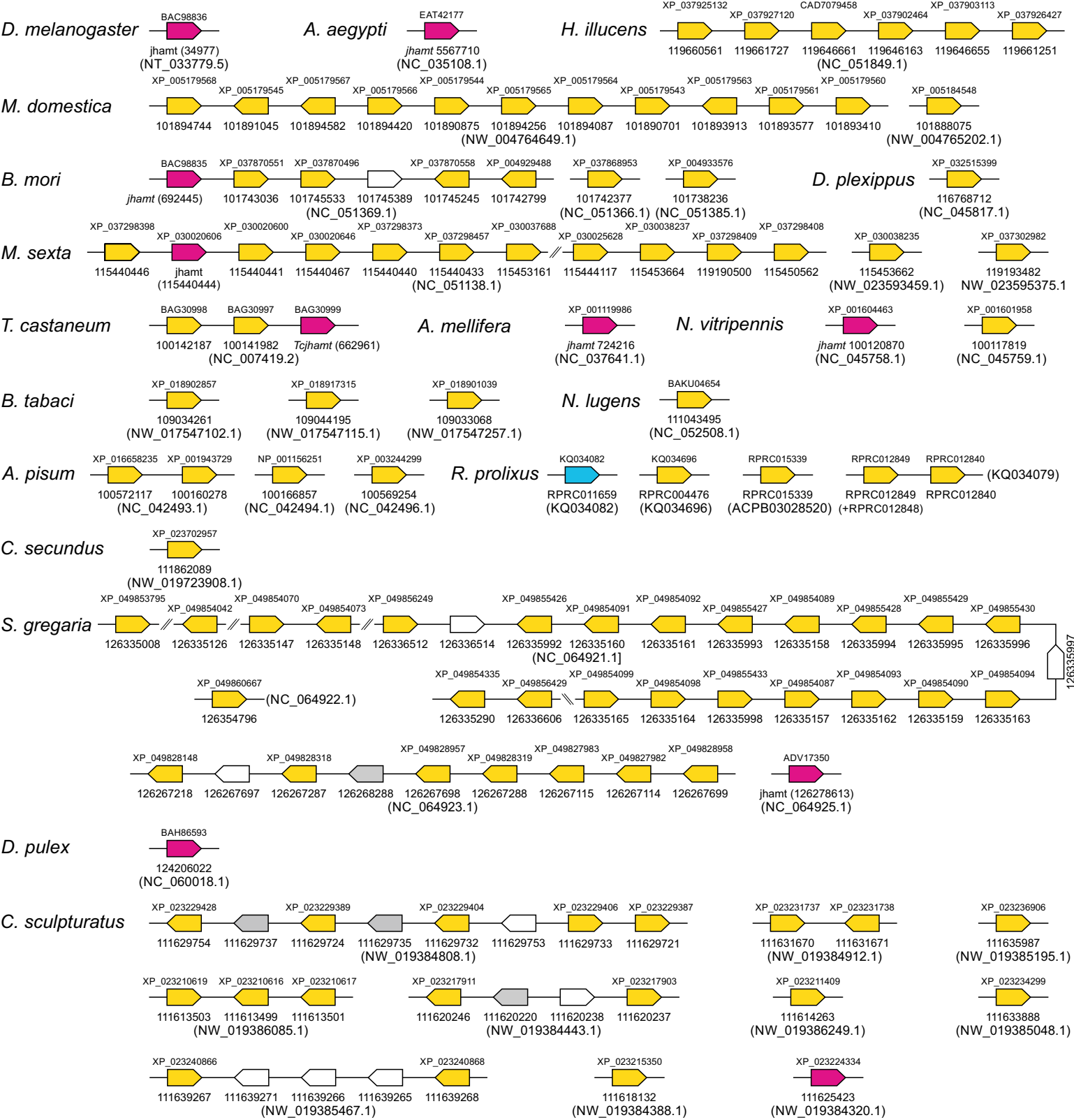

Supplement: Supplementary data 2 [file mmc2.pdf]

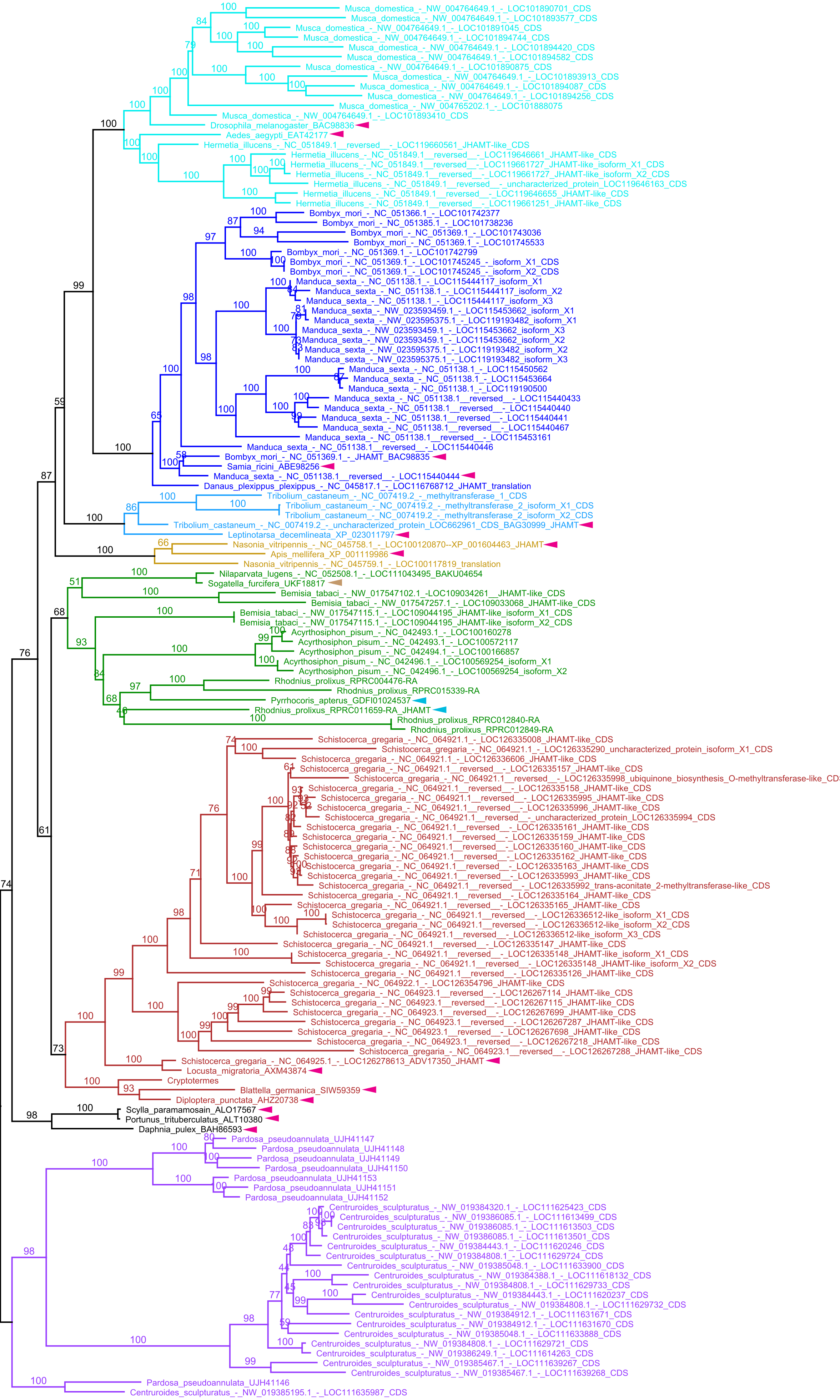

Supplement: Supplementary data 3 [file mmc3.pdf]

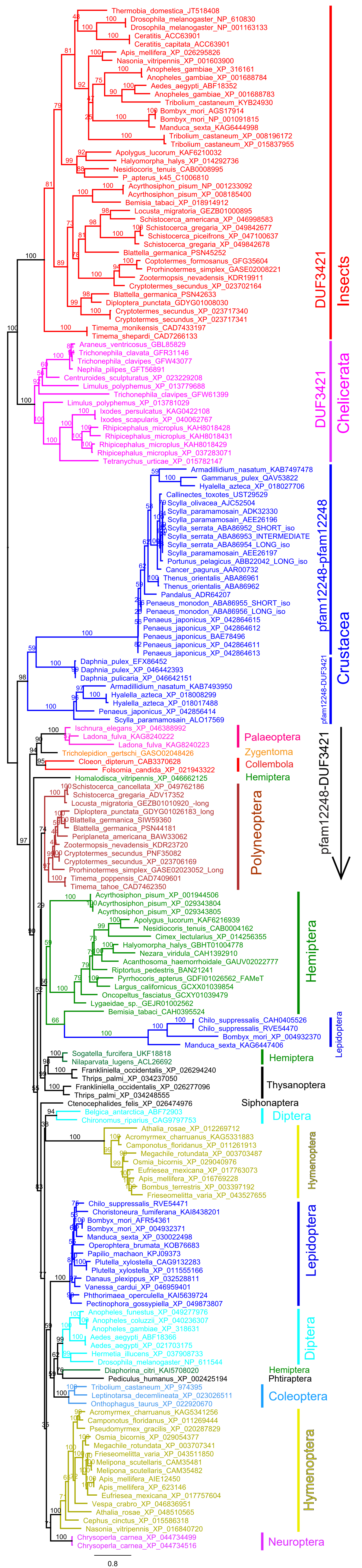

Supplement: Supplementary data 4 [file mmc4.pdf]

*Penaeus japonicus* BAE78496

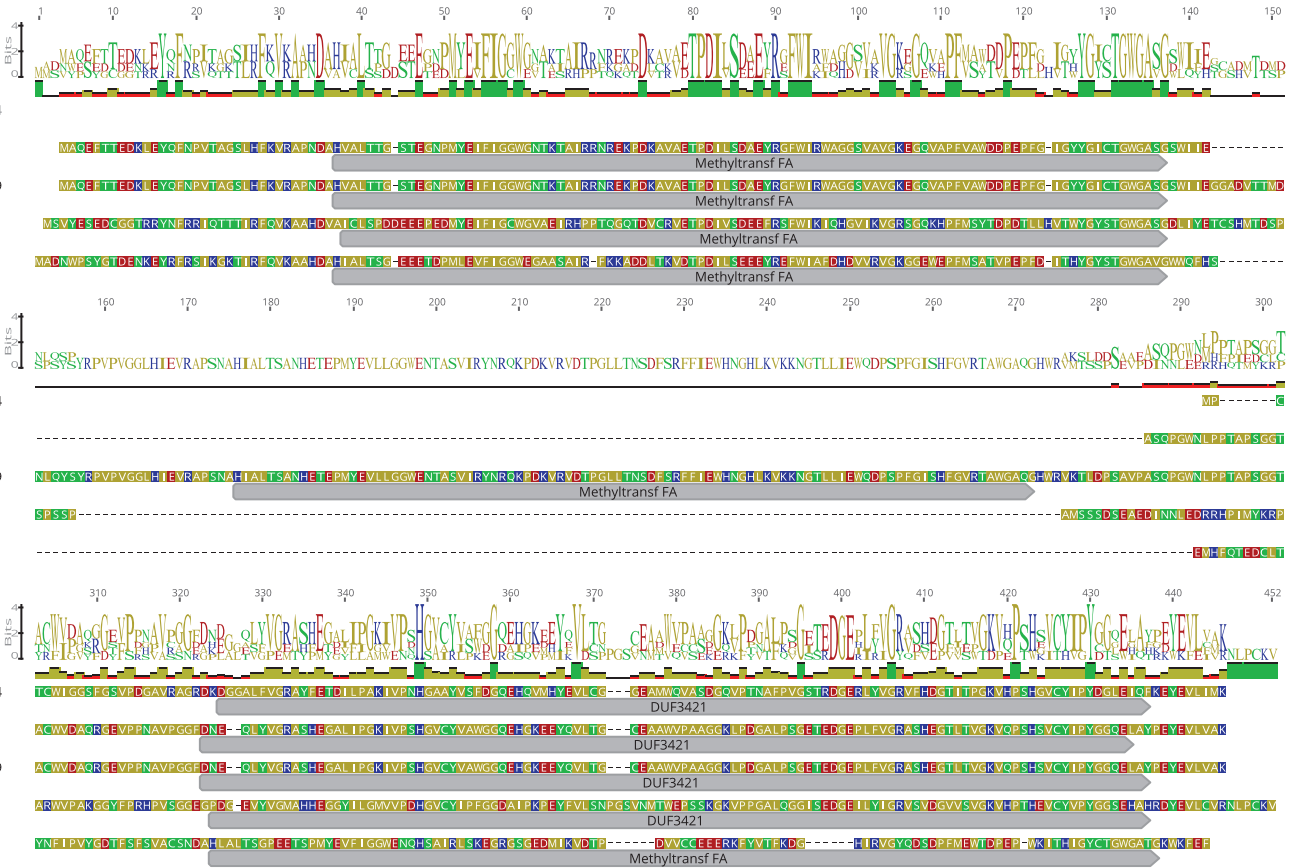

Supplement: Supplementary data 5 [file mmc5.pdf]

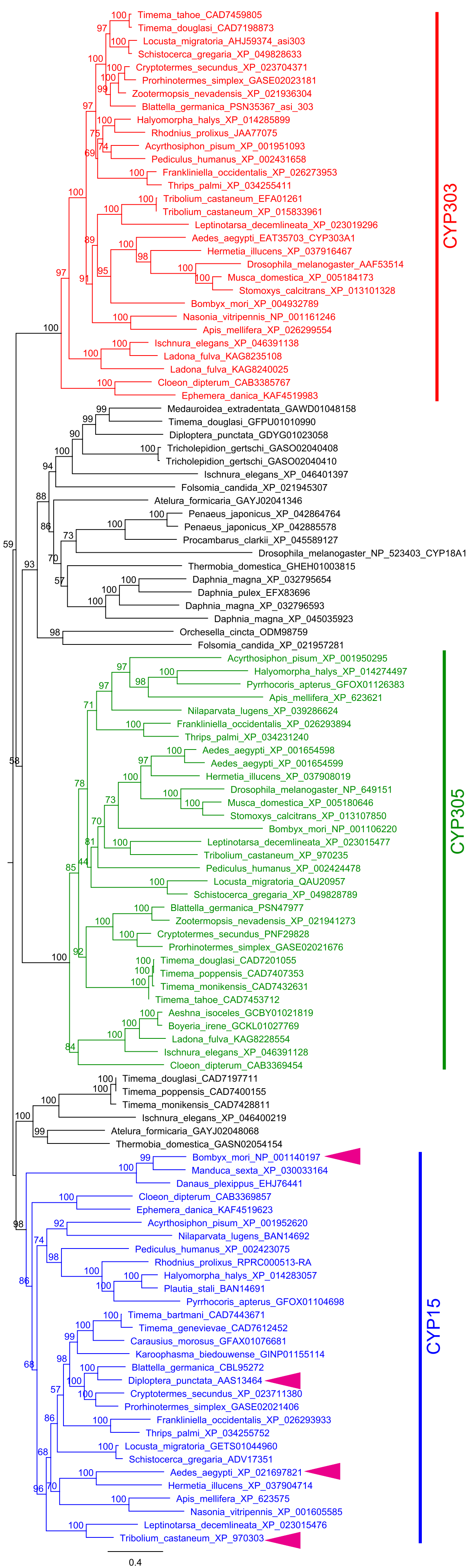

Supplement: Supplementary data 6 [file mmc6.pdf]
