# Supplementary material for: Evolution of proteins involved in the final steps of juvenile hormone synthesis
Source: J Insect Physiol. 2023 Mar;145:104487. doi: 10.1016/j.jinsphys.2023.104487 (PMC10015273; doi:10.1016/j.jinsphys.2023.104487)
Supplement: Supplementary data 7 [file mmc7.docx]

**Supportive material for Fig. 7**

References, reporting direct determination of Methyl Farnesoate and/or Juvenile hormone(s) in insect and arthropod species.

**Diptera**

***Drosophila melanogaster***

Methyl Farnesoate (MF)

Tu et al., 2005, GCE, 10.1016/j.ygcen.2005.02.009

Jones et al., 2013, GCE, 10.1016/j.ygcen.2012.11.009

Juvenile Hormone III (JH-III)

Sliter et al., 1987, Insect Biochem, 10.1016/0020-1790(87)90156-9

Bownes and Rembold, 1987, Eur J Biochem, 10.1111/j.1432-1033.1987.tb11184.x

Tu et al., 2005, GCE, 10.1016/j.ygcen.2005.02.009

Jones et al., 2013, GCE, 10.1016/j.ygcen.2012.11.009

Ramirez et al., 2020, IBMB, 10.1016/j.ibmb.2019.103287

Juvenile Hormone III bisepoxide (JHB3)

Richard et al., 1989a, PNAS, 10.1073/pnas.86.4.1421

Richard et al., 1989b, J Comp Physiol B, 10.1007/BF00692410

Tu et al., 2005, GCE, 10.1016/j.ygcen.2005.02.009

Jones et al., 2013, GCE, 10.1016/j.ygcen.2012.11.009

Ramirez et al., 2020, IBMB, 10.1016/j.ibmb.2019.103287

***Musca domestica***

Methyl Farnesoate (MF)

Teal et al., 2014, J. Nat. Prod., 10.1021/np400807v

Juvenile Hormone I (JH-I) – The presence of JH-I has not been confirmed since then.

Girard et al., 1976, Insect Biochem, 10.1016/0020-1790(76)90034-2

Schooley et al., 1976, p. 101-117. In: Gilbert, L.I. (eds) The Juvenile Hormones. Springer, Boston, MA. 10.1007/978-1-4684-7947-8_8

Juvenile Hormone II (JH-II) – The presence of JH-II has not been confirmed since then.

Schooley et al., 1976, p. 101-117. In: Gilbert, L.I. (eds) The Juvenile Hormones. Springer, Boston, MA. 10.1007/978-1-4684-7947-8_8

Juvenile Hormone III (JH-III)

Schooley et al., 1976, p. 101-117. In: Gilbert, L.I. (eds) The Juvenile Hormones. Springer, Boston, MA. 10.1007/978-1-4684-7947-8_8

Teal et al., 2014, J. Nat. Prod., 10.1021/np400807v

Juvenile Hormone III bisepoxide (JHB3)

Teal et al., 2014, J. Nat. Prod., 10.1021/np400807v

***Aedes aegypti***

Methyl Farnesoate (MF)

Jones et al., 2010, JIP; 10.1016/j.jinsphys.2010.06.001

Juvenile Hormone I (JH-I) – The presence of JH-I has not been confirmed since then.

Borovsky et al., 1994, IBMB, 10.1016/0965-1748(94)90038-8

Juvenile Hormone III (JH-III)

Shapiro et al., 1986, JIP, 10.1016/0022-1910(86)90102-2

Ramirez et al., 2020, IBMB, 10.1016/j.ibmb.2019.103287

Juvenile Hormone III bisepoxide (JHB3) – The presence of JH-I has not been confirmed since then.

Borovsky et al., 1994, IBMB, 10.1016/0965-1748(94)90038-8

**Lepidoptera**

***Bombyx mori***

Juvenile Hormone I (JH-I)

Furuta et al., 2013, BBB, 10.1271/bbb.120883

Ramirez et al., 2020, IBMB, 10.1016/j.ibmb.2019.103287

Juvenile Hormone II (JH-II)

Furuta et al., 2013, BBB, 10.1271/bbb.120883

Ramirez et al., 2020, IBMB, 10.1016/j.ibmb.2019.103287

Juvenile Hormone III (JH-III)

Furuta et al., 2013, BBB, 10.1271/bbb.120883

Ramirez et al., 2020, IBMB, 10.1016/j.ibmb.2019.103287

***Manduca sexta***

Juvenile Hormone O (JH-O)

Bergot et al., 1980, Science, 10.1126/science.210.4467.336

Bergot et al., 1981, J Chrom A, 10.1016/S0021-9673(00)81664-7

Goodman, W.G., Granger, N.A., 2005. The juvenile hormones. In: Gilbert, L.I., Iatrou, K., Gill, S.S. (Eds.), Comprehensive Molecular Insect Science, vol. 3. Elsevier Ltd., Oxford, pp. 319–408.

Juvenile Hormone I (JH-I)

Bergot et al., 1981, J Chrom A, 10.1016/S0021-9673(00)81664-7

Ramirez et al., 2020, IBMB, 10.1016/j.ibmb.2019.103287

Juvenile Hormone II (JH-II)

Judy et al., 1973, PNAS, 10.1073/pnas.70.5.1509

Bergot et al., 1981, J Chrom A, 10.1016/S0021-9673(00)81664-7

Ramirez et al., 2020, IBMB, 10.1016/j.ibmb.2019.103287

Juvenile Hormone III (JH-III)

Judy et al., 1973, PNAS, 10.1073/pnas.70.5.1509

Bergot et al., 1981, J Chrom A, 10.1016/S0021-9673(00)81664-7

4-methyl Juvenile Hormone I (4-methyl JH-I)

Bergot et al., 1981. In G. E. Pratt, & G.

T. Brooks (Eds.), Juvenile Hormone Biochemistry (pp. 33–45).

**Coleoptera**

***Phyllophaga crinita, Prionus imbricornis, Aethina tumida***

Methyl Farnesoate (MF)

Teal et al., 2014, J. Nat. Prod., 10.1021/np400807v

***Tribolium castaneum***

Juvenile Hormone III (JH-III)

Parthasarathy et al., 2009, Mech. Dev., 10.1016/j.mod.2009.03.00

Parthasarathy et al., 2010, IBMB, 10.1016/j.ibmb.2010.03.006

***Tenebrio molitor***

Juvenile Hormone III (JH-III)

Trautmann et al., Z. Naturforsch C, 1974, 10.1515/znc-1974-11-1218

Judy et al., Life Sci, 1975, 10.1016/0024-3205(75)90190-3

**Hymenoptera**

***Apis mellifera***

Methyl Farnesoate (MF)

Teal et al., 2014, J. Nat. Prod., 10.1021/np400807v

Juvenile Hormone III (JH-III)

Trautmann et al., Z. Naturforsch C, 1974, 10.1515/znc-1974-11-1218

Rembold, 1987, Insect Biochem, 10.1016/0020-1790(87)90110-7

Teal et al., 2014, J. Nat. Prod., 10.1021/np400807v

**Hemiptera: Sternorrhyncha**

***Acyrthosiphon pisum***

Juvenile Hormone III (JH-III)

Chen et al., 2007, IBMB, 10.1016/j.ibmb.2007.05.019

**Hemiptera: Heteroptera**

Methyl Farnesoate (MF)

Teal et al., 2014, J. Nat. Prod., 10.1021/np400807v

Juvenile Hormone III (JH-III) – Although Teal et al., 2014 claim detection of JH III, they measured only MF, JH III, and JHB3, but not JHSB3!

Teal et al., 2014, J. Nat. Prod., 10.1021/np400807v

***Pyrrhocoris apterus***

Juvenile Hormone III skipped bisepoxide (JHSB3)

Hejnikova et al., 2016, JIP, 10.1016/j.jinsphys.2016.08.009

Hejnikova et al., 2022, IBMB, 10.1016/j.ibmb.2022.103721

***Rhodnius prolixus***

Juvenile Hormone III skipped bisepoxide (JHSB3)

Villalobos-Sambucaro et al., 2020, Sci Rep. 10.1038/s41598-020-59495-1

Juvenile Hormone I, II, III (JH-I, JH-II, JH-III) – A detection of listed JHs was most likely a result of cross-reacting Radio-Immune Assay JH-specific antibodies. JHSB3 was not known at the time.

Baehr, J. C., Porcheron, P. & Dray, F. 1978 Dosages radio-immunologiques des hormones juveniles au tours des deux derniers stades larvaires de Rhodnius prolixus. C. R. H. S. Acad. Sci., Paris. 281, 523–526.

**Polyneoptera: Orthoptera**

***Schistocerca americana***

Methyl Farnesoate (MF)

Teal et al., 2014, J. Nat. Prod., 10.1021/np400807v

***Schistocerca gregaria***

Juvenile Hormone III (JH-III)

Pratt and Tobe, Life Sci, 1974, 10.1016/0024-3205(74)90372-5

Trautmann et al., Z. Naturforsch C, 1974, 10.1515/znc-1974-11-1218

Blight and Wenham, 1976, Insect Biochem, 10.1016/0020-1790(76)90056-1

***Schistocerca vaga***

Juvenile Hormone III (JH-III)

Judy et al., Life Sci, 1973, 10.1016/0024-3205(73)90139-2

***Locusta migratoria***

Juvenile hormone I (JH-I)

Baehr et al., JIP, 1979, 10.1016/0022-1910(79)90009-X) – A detection of JH-I was most likely a result of cross-reacting Radio-Immune Assay JH-specific antibodies.

Juvenile Hormone III (JH-III)

Huibregtse-Minderhoud et al., 1980, JIP, 10.1016/0022-1910(80)90032-3

Bergot et al., 1981, Experientia, 10.1007/BF01985709

Dale and Tobe, 1986, JIP, 10.1016/0022-1910(86)90079-X

**Polyneoptera: Blattodea**

***Blattella germanica***

Juvenile Hormone III (JH-III)

Camps et al., 1987, Arch. Insect Biochem. Physiol., 10.1002/arch.940060306

Treiblmayr et al., 2006, JIS, 10.1673/031.006.4301

***Diploptera punctata***

Juvenile Hormone III (JH-III)

Holbrook et al., 1998, Invert Rep and Dev, 10.1080/07924259.1998.9652343

Stay et al., 2002, Peptides, 10.1016/S0196-9781(02)00185-7

Methyl Farnesoate (MF)

Neese et al., 2000, JIP, 10.1016/s0022-1910(99)00134-1

Stay et al., 2002, Peptides, 10.1016/S0196-9781(02)00185-7

***Nauphoeta cinerea***

Juvenile Hormone I, -II, -III (JH-I, JH-II, JH-III) – The presence of JH-I and JH-II has not been confirmed since then.

Lanzrein et al., Life Sci, 1975, 10.1016/0024-3205(75)90312-4

***Cryptotermes secundus***

Juvenile Hormone III (JH-III)

Korb et al., 2012, JIP, 10.1016/j.jinsphys.2011.12.016

**Crustacea**

***Procambarus clarkii***

Methyl Farnesoate (MF)

Kwok et al., 2005, JIP, 10.1016/j.jinsphys.2004.12.010

***Libinia emarginata***

Methyl Farnesoate (MF)

Laufer et al., 1987, Science, 10.1126/science.235.4785.202

Laufer et al., 1987, Insect Bochem, 10.1016/0020-1790(87)90134-X

Borst et al, 1987, Insect Biochem, 10.1016/0020-1790(87)90133-8

***Homarus americanus***

Methyl Farnesoate (MF)

Borst et al, 1987, Insect Biochem, 10.1016/0020-1790(87)90133-8

***Daphnia pulex***

No Methyl Farnesoate (MF) detected

Toyota et al., 2015, JIP, 10.1016/j.jinsphys.2015.02.002

**Chelicerata: Acari**

***Dermacentor variabilis* and** ***Ornithodoros parkeri***

No Methyl Farnesoate (MF) detected

Neese et al., 2000, JIP, 10.1016/s0022-1910(99)00134-1
